# Probing the Interfacial Behavior of Type IIIa Binary Mixtures Along the Three-Phase Line Employing Molecular Thermodynamics

**DOI:** 10.3390/molecules25071499

**Published:** 2020-03-25

**Authors:** Gerard Alonso, Gustavo Chaparro, Marcela Cartes, Erich A. Müller, Andrés Mejía

**Affiliations:** 1 Departament de Ciència de Materials i Química Física & Institut de Química Teòrica i Computacional (IQTCUB), Universitat de Barcelona, 08028 Barcelona, Spain; g.alonso@ub.edu; 2 Departamento de Ingeniería Química, Universidad de Concepción POB 160–C, Concepción, Chile; gustavochaparro@udec.cl (G.C.); marcecartes@udec.cl (M.C.); 3 Department of Chemical Engineering, Imperial College London, South Kensington Campus, London SW7 2AZ, UK; e.muller@imperial.ac.uk

**Keywords:** square gradient theory, molecular dynamics, aqueous-hydrocarbon mixture, three-phase line interfacial properties, SAFT-VR Mie

## Abstract

Interfacial properties such as interfacial profiles, surface activity, wetting transitions, and interfacial tensions along the three-phase line are described for a Type IIIa binary mixture. The methodological approach combines the square gradient theory coupled to the statistical associating fluid theory for Mie potentials of variable range, and coarse-grained molecular dynamics simulations using the same underlying potential. The water + *n*-hexane mixture at three-phase equilibrium is chosen as a benchmark test case. The results show that the use of the same molecular representation for both the theory and the simulations provides a complementary picture of the aforementioned mixture, with an excellent agreement between the molecular models and the available experimental data. Interfacial tension calculations are extended to temperatures where experimental data are not available. From these extrapolations, it is possible to infer a first order wetting transition at 347.2 K, where hexane starts to completely wet the water/vapor interface. Similarly, the upper critical end point is estimated at 486.3 K. Both results show a very good agreement to the available experimental information. The concentration profiles confirm the wetting behavior of *n*-hexane along with a strong positive surface activity that increases with temperature, contrasting the weak positive surface activity of water that decreases with temperature.

## 1. Introduction

**Figure 1:**
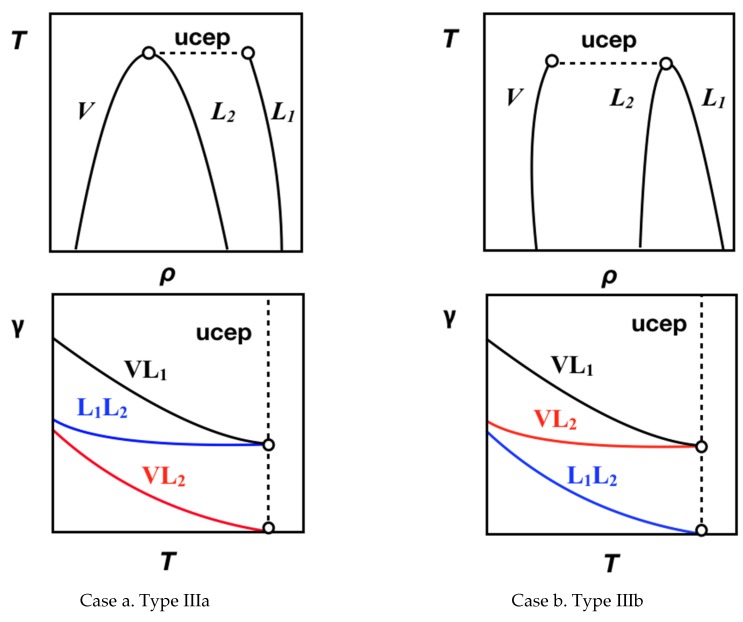
Schematic representation of Temperature, *T*—Density, *ρ* and Interfacial Tension, *γ*—Temperature, *T* diagrams for three-phase equilibrium in Type III binary mixture. Case a: Type IIIa; Case b. Type IIIb.

According to the van Konynenburg and Scott [1] classification of phase equilibria, Type III mixtures are characterized by a branch of the critical line ending in an upper critical end point (UCEP) of a three-phase line, while the other branch diverges to the high-pressure range. This implies that at subcritical conditions, mixtures of this type can display vapor–liquid (*VL_1_* or *VL_2_*), liquid–liquid (*L_1_L_2_*), and vapor–liquid–liquid (*VL_1_L_2_*) equilibria. For these mixtures, the interplay between bulk phases and their interfaces constitutes a challenging task for applied thermodynamics due to the sharp asymmetry between the intermolecular forces at bulk and interfacial regions. One of the prototypical examples of this Type III equilibria are the water + *n*-alkane [*n* ≥ 2] mixtures, where the relation between bulk phases and their interfaces is also a function of the *n*-alkane molecular chain length (*n*). Specifically, for *n* = 2 to 26, the critical line connecting the critical point of the *n*-alkane ends in an UCEP characterized by a vapor–liquid critical point and an aqueous-rich liquid phase; this behavior is classified as Type IIIa. For *n* = 28 to 32, the critical line connecting the critical point of pure water ends in an UCEP characterized by a vapor–liquid critical point and an *n*-alkane-rich liquid phase; this behavior is classified as Type IIIb. Finally, 26 < *n* < 28, water + *n*-alkane mixtures display a mass density (or barotropic) inversion, where the relative position of immiscible phases changes. Figure 1 displays a schematic representation of the coexistent density of Types IIIa and IIIb_._ (see Ref. [2] for further details on critical lines and subcritical phase equilibria for water + *n*-alkane [*n* ≥ 2] mixtures).

Additional to water + *n*-alkane mixtures, the phase equilibria and interfacial properties for other Type III mixtures, such as carbon dioxide and hexadecane mixture and mixtures containing polymers, have been explored by Virnau et al. [3] and Müller et al. [4], respectively.

Figure 1 suggest that the interfacial behavior is governed by the localization of the different bulk phase densities. Considering the possible interfaces (*VL_1_*,*VL_2_*, *L_1_L_2_*), three interfacial tensions (IFTs) can be defined: *γ^VL1^*, *γ^VL2^*, and *γ^L1L2^*, where *L_1_* refers to the water rich and *L_2_* to the *n*-alkane rich liquid phases, and *V* is the vapor phase at equilibrium. Assuming for instance that *γ^VL1^* > *γ^L1L2^* > *γ^VL2^* (case a, Figure 1), these IFTs are interrelated by the expression [5]: *γ^VL1^* ≤ *γ^L1L2^ + γ^VL2^*. The equality denotes the situation of total (or perfect) wetting of the *L_2_* bulk phase in *VL_1_* interface (Antonow’s rule), whereas the inequality describes the situation of partial wetting of the *L_2_* bulk phase in *VL_1_* interface (Neumann inequality). The transition from total to partial wetting (or vice versa) is recognized as a wetting transition [6] and occurs at a certain point along the three-phase line. (see Refs. [7,8] and references therein). Additionally, when temperature increases and approaches the UCEP, the densities of two bulk phases becomes equal, *ρ^L2^* ≈ *ρ^V^* for Type IIIa (case a, Figure 1), or *ρ^L1^* ≈ *ρ^L2^* for Type IIIb (case b, Figure 1) causing that the interfacial tensions at the UCEP to approach *γ^VL1^* = *γ^L1L2^* and *γ^VL2^* = 0 or *γ^VL1^* = *γ^VL2^* and *γ^L1L2^* = 0, respectively.

To characterize the interfacial properties of the water + *n*-alkane mixtures along three-phase line, a combination of experimental determinations, theoretical approaches, and molecular dynamics simulations can be used. This schema allows a global and unambiguous understanding of the interfacial properties (e.g., concentration profiles along the interfacial region, surface activity of all species, interfacial tensions), wetting transitions, UCEP temperature, and their relationship with the three-phase equilibrium.

From an experimental point of view, the pendant drop technique (see Ref. [9,10] and references therein) is the most appropriate technique available. In this method, a liquid phase drop (*L_1_* or *L_2_*) is suspended on the tip of a needle. This drop is immersed in the other phase (be it *L_2_* or *L_1_* or *V*) in a closed chamber where temperature and/or pressure can be controlled. Interchanging the liquid of the drop and its surroundings, it is possible to independently measure the three interfacial tensions (*γ^VL1^*; *γ^VL2^*; *γ^L1L2^*). This tensiometric technique has been used by Mori et al., [11] for measuring some Type IIIa of water + *n*-alkane mixtures, where *γ^VL1^* > *γ^L1L2^* > *γ^VL2^*.

Our theoretical approach is based on the square gradient theory (SGT) [12,13], where the inhomogeneous behavior is described by a Taylor expansion of the Helmholtz energy density around the homogeneous state. The final expression of this continuous Helmholtz energy density has been successfully used to characterize three-phase equilibrium and interfacial behavior such as interfacial concentration profiles, interfacial tensions and wetting in both conceptual model fluids [5,14,15,16,17,18,19] and experimental [20] fluids. Alternatively to SGT, the self-consistent field theory has been used to describe both three-phase equilibrium and interfacial behavior in Type III mixtures (see Ref. [4] and references therein).

Molecular Dynamics (MD) and Monte Carlo (MC) molecular simulations of the mixtures of vapor–liquid–liquid equilibria (VLLE) can be performed in the canonical, *NVT*, or the *NP_zz_AT* ensembles. In MD, the interfacial tension is calculated by the Hulshof integral [21], where the pressure along the interfacial region is described by the Irving–Kirkwood (IK) tensor [22]. This approach has been successfully used by us in previous works to describe the three-phase interfacial tension for Lennard–Jones [18,19,23], and coarse-grained Mie [24] fluids. In the case of MC, the interfacial tension can be calculated using similar approach or alternatively using the grand canonical ensemble (*μ, T, V*). In the grand canonical ensemble, the interfacial tension is calculated by using the average height of the peaks of the probability distribution as a function of the number of particles together with the definition of interfacial free energy. For further details concerning to this methodology, the reader is redirected to Ref. [3] and references therein.

The description of the interfacial properties of water + *n*-alkane mixtures by simultaneously using experiments, theory and simulations is not always possible along the entire three-phase line. Specifically, the main limitation of the experimental determinations are the normal boiling temperature of fluids (*T_NB_*) which are significantly lower than the UCEP temperatures (*T_UCEP_*). Therefore, it is necessary to use alternative procedures (theory and/or simulation) to fill the gap from *T_NB_* to *T_UCEP_*. On the other hand, the SGT coupled to any equation of state is usually unable to predict interfacial tensions in liquid-liquid equilibrium without input from experimental data. The reader is referred to the extensive digressions by Carey [13] and the implementations by Cornelisse et al. [20] in selected systems (water + *n*-hexane, water + benzene and *n*-hexane + water + ethanol mixtures). Consequently, SGT needs to use the interfacial tension data of the mixture to fine-tune the description of the interfacial behavior in liquid-liquid and liquid-liquid-vapor equilibrium. In contrast, MD simulations are capable of predicting interfacial properties via explicit simulation of the vapor-liquid-liquid (VLL) interfaces, where the required force field parameters can be parametrized by the use of pure component thermophysical and mixture phase equilibria data rather than having to recourse to interfacial tension data.

In this work, we select the water + *n*-hexane binary mixture along the three-phase line to exemplify the interfacial properties for Type IIIa systems. In this case, the available tensiometry data [11] only cover the temperature range from 293.15 K to 333.15 K. This maximum temperature is capped by the normal boiling temperature of *n*-hexane (*T_NB_* = 341.88 K [25]) which is relative low in comparison to *T_UCEP_* = 495.82 K [26]. Additionally, Bertrand et al., [27] reported an experimental wetting transition at *T_w_* = 345.4 K, which was verified by Cornelisse et al. [20] by employing the Peng-Robinson EoS with SGT, but not seen with the APACT EoS.

To explore the interfacial behavior from 333.15 K to 495.82 K, we use here both theory and simulations. MD results additionally be used to verify the SGT results. To carry out this validation, we select the Coarse-Grained Mie potential [28,29] to model pure fluids and the fluid mixture. The main two advantages of this force field are: (*i*) its capacity of describing simultaneously bulk phases and interfaces and (*ii*) the possibility of using it in both MD and SGT coupled to the statistical associating fluid theory for Mie potentials of variable range (SAFT-VR Mie) EoS [30] to obtain a complementary picture of the experimentally inaccessible interfacial phenomena.

The rest of the paper is organized as follows: In Section 2, we summarize the main expressions of the SGT and the SAFT-VR Mie EoS. In Section 3, we describe the MD simulation techniques used to calculate the properties of interest. Lastly, in Section 4, we present and discuss the interfacial properties obtained from theory and simulations, as well as their comparison to experimental data. In Section 5 we draw the main conclusions of this work.

## 2. Theory

### 2.1. Square Gradient Theory for Mixtures

The modeling of interfacial tension for fluid mixtures is carried out applying the SGT [12,13] coupled to SAFT-VR Mie EoS [30] as a model to describe the homogenous part of the interfacial Helmholtz energy density. Following the SGT, the isothermal interfacial tension (*γ*) of mixtures arises from the boundary of two bulk phases in equilibrium, *α β* (here *αβ* represents *VL_1_*; *VL_2_* or *L_1_L_2_*), and is given by the following integral expression [13]:

(1) $$\gamma^{\alpha \beta }=\int_{z_{\alpha }}^{z_{\beta }}\sum_{i,j=1}^{n_{c}}\kappa_{ij}\sqrt{c_{ii}c_{jj}}\left(\frac{d\rho_{i}}{dz}\right)\left(\frac{d\rho_{j}}{dz}\right)dz$$

where *n_c_* is the number of species (*n_c_* = 2 in this work), *ρ_i_* is the interfacial molar concentration of species *i* and *z* is the spatial coordinate perpendicular to the planar interface. The superscripts *α* and *β* correspond to the two different bulk phases. *c_ii_* is the influence parameter of the pure fluid *i*. *κ_ij_* is a symmetric cross interfacial tension parameter for the mixture (*κ_ij_* = *κ_ji_*; *κ_ii_* = *κ_jj_* = 1), which is obtained by fitting Equation (1) with the experimental IFT values of the mixture.

In this work, *c_ii_* is calculated from the pure fluid coarse-grained (CG)-Mie parameters using the following expression [31]:

(2) $$\sqrt{\frac{c_{ii}}{N_{av}^{2}\varepsilon_{ii}\sigma_{ii}^{5}}}=m_{si}\left(0.12008+2.21979\alpha_{ii}\right)$$

In this expression, *N_av_* is the Avogadro’s constant, *ε_ii_* is the energy scale corresponding to the Mie potential well depth, *σ_ii_* length scale, corresponding with an effective segment diameter of the Mie potential, *m_si_* is the molecular chain length for the pure fluid *i* described by the CG approach, and *α_ii_* is the van der Waals constant given by:

(3) $$\alpha_{ii}=C_{ii}\left[\left(\frac{1}{\lambda_{a,ii}-3}\right)-\left(\frac{1}{\lambda_{r,ii}-3}\right)\right]$$

where *λ_r,ii_* and *λ_a,ii_* are the repulsion and attraction parameters of the intermolecular (Mie) potential for pure fluid *i*, respectively. *C_ii_* is a constant, which is defined as:

(4) $$C_{ii}=\frac{\lambda_{r,ii}}{\lambda_{r,ii}-\lambda_{a,ii}}{\left(\frac{\lambda_{r,ii}}{\lambda_{a,ii}}\right)}^{\frac{\lambda_{a,ii}}{\lambda_{r,ii}-\lambda_{a,ii}}}$$

Table 1 summarizes the numerical values of the Mie potential for pure fluids, which are also used to derive the *c_ii_* values through Equation 2. These values will be simultaneously used in theoretical calculation and molecular simulations to obtain phase equilibrium and interfacial properties.

In the framework of the SGT, *ρ_i_* (*z*) is calculated by solving the following system of differential equations [13]:

(5) $$\overset{n_{c}}{\underset{j=1}{\sum }}\kappa_{ij}\sqrt{c_{ii}c_{jj}}\frac{d^{2}\rho_{j}}{dz^{2}}=-\frac{\partial }{\partial \rho_{i}}\left[a_{0}-\overset{n_{c}}{\underset{i=1}{\sum }}\rho_{i}\mu_{i}^{0}\right]\;\;\;i=1,2,\cdots ,n_{c}\;\;\;\{\begin{array}{c} {\rho_{i}|}_{z=z^{\alpha }}=\rho_{i}^{\alpha }\\ {\rho_{i}|}_{z=z^{\beta }}=\rho_{i}^{\beta } \end{array}$$

In Equation (5), *a_0_* is the Helmholtz energy density of the homogenous system, which is given by the SAFT-VR Mie EoS for non-associating chain fluids [30], *μ_i_^0^* is the chemical potential of species *i* evaluated at the phase equilibrium conditions, calculated from its definition in the canonical ensemble *μ_i_^0^* = (∂*a_0_/*∂*ρ_i_*)*_T,V,__ρj_*.

**Figure 2:**
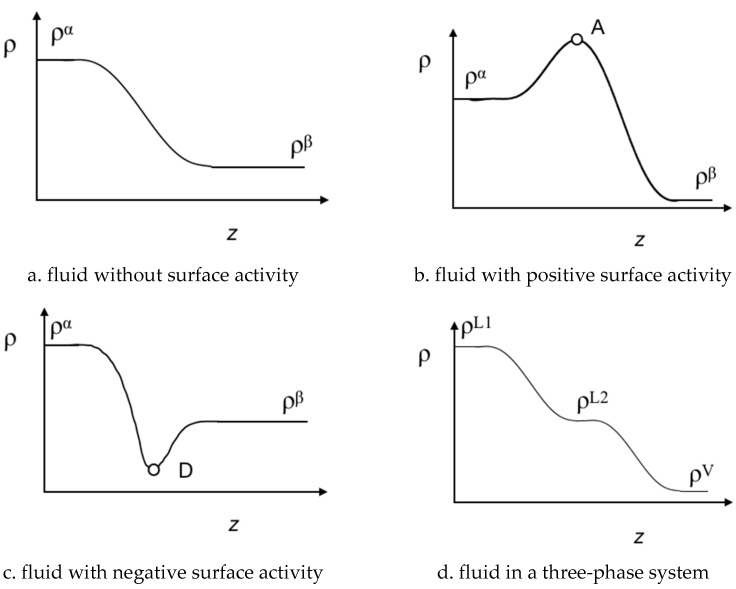
Schematic representation of the multiphasic interfacial concentration profiles, *ρ_i_*, as a function of the *z* coordinate. (**a**) pure fluids or fluid in mixtures without surface activity in a biphasic system; (**b**) fluids with a positive surface activity (adsorption) in a biphasic system; (**c**) fluids with a negative surface activity (desorption) in a biphasic system; (**d**) fluids without surface activity in a triphasic system.

Interfacial profiles for the species, *ρ_i_* (*z*) are obtained solving Equation (5), from which the interfacial tension can be calculated using Equation (1). Furthermore, with the information of the *ρ_i_* (*z*) profiles, it is possible to characterize the surface activity of the species along the interfacial region. Figure 2 illustrates four possible patterns of *ρ_i_* (*z*). Figure 2a shows the common biphasic interfacial profile for pure fluids or fluid mixtures without surface activity. In Figure 2b,c, the interfacial profile displays a stationary point, which can be a maximum (point A) or a minimum (point D). The stationary points reflect adsorption (A) or desorption (D) of species along the interface region (i.e., the surface activity). Finally, Figure 2d displays a possible density profile *ρ_i_* (*z*) for *VL_1_L_2_* equilibrium. In the latter, the interfacial concentration along the *z* coordinate shows three interfaces without surface activity, namely *VL_1_*, *L_1_L_2_*, *VL_2_*. The *ρ_i_* (*z*) profile also provides a route to explore the interfacial concentration of the mixture and its thermal evolution for three-phase systems. As illustrated in Figure 1 and reported in previous works (see Refs. [16,17,18,19]) *ρ^L1^*(*z*) ≠ *ρ^L2^* (*z*) + *ρ^V^* (*z*) at *T* < *T_w_*, whereas *ρ^L1^*(*z*) = *ρ^L2^* (*z*) + *ρ^V^* (*z*) at *T* > *T_w_*. For the case that *T* → *T_UCEP_*, *ρ^L2^*(*z*) ≈ *ρ^V^* (*z*) or *ρ^L2^*(*z*) ≈ *ρ^L1^* (*z*).

### 2.2. The Statistical Associating Fluid Theory Model

The SAFT-VR Mie is a particular case of the generic SAFT methodology for potentials of variable range, which represents molecules conformed of segments interacting through the Mie potential [32], *u^Mie^*, represented by:

(6) $$u^{Mie}\left(r_{ij}\right)=C_{ij}\varepsilon_{ij}\left[{\left(\frac{\sigma_{ij}}{r_{ij}}\right)}^{\lambda_{r,ij}}-{\left(\frac{\sigma_{ij}}{r_{ij}}\right)}^{\lambda_{a,ij}}\right]$$

In this expression, *r_ij_* is the center-to-center distance of the interacting segments. The other terms have the same meaning described before but extended to mixtures applying combination rules [30]. Specifically, the unlike size parameter, *σ_ij_* is obtained using an arithmetic mean:

(7) $$\sigma_{ij}=\left(\sigma_{ii}+\sigma_{jj}\right)/2$$

while the unlike Mie attractive interaction energy (or cross potential well depth), *ε_ij_* is obtained using a Berthelot-like geometric average:

(8) $$\varepsilon_{ij}=\left(1-k_{ij}\right)\frac{\sqrt{\sigma_{ii}^{3}\sigma_{jj}^{3}}}{\sigma_{ij}^{3}}\sqrt{\varepsilon_{ii}\varepsilon_{jj}}$$

where *k_ij_* is a binary interaction parameter, which is obtained from experimental data of phase equilibria. The cross attractive (*λ_a,ij_*) and repulsive (*λ,_rij_*) parameters involved in the Mie potential are calculated as:

(9) $$\left(\lambda_{k,ij}-3\right)=\sqrt{\left(\lambda_{k,ii}-3\right)\left(\lambda_{k,jj}-3\right)}\;\;\;;\;\;k=a,r$$

The prefactor *C_ij_* is given in Equation (4), where the attractive and repulsive exponents are replaced by *λ_a,ij_* and *λ,_rij_*, respectively. Finally, the homogeneous Helmholtz energy density for the SAFT-VR Mie EoS for non-associating chain fluid is given by [30]:

(10) $$a_{0}=\left(a^{mono}+a^{chain}+a^{ig}\right)\rho N_{av}/\beta$$

where *a_0_* = *A*/(*N k_B_ T*), *A* being the total Helmholtz energy, *N* the total number of molecules, *N_av_* the Avogadro constant, *T* the temperature, *k_B_* the Boltzmann constant, *β* = 1/(*k_B_T*), and *ρ* the molar density of the mixture. *a^mono^* is the Helmholtz energy contribution of the unbounded monomers forming a chain of *m_s_* tangential segments, *a^chain^* accounts for the Helmholtz energy of chain formation, and *a^ig^* is the Helmholtz energy of the perfect gas reference.

To summarize, the key parameters involved in the SAFT-VR Mie EoS are the pure component parameters (*m_si_, λ_r,ii_, λ_a,ii,_ ε_ii_, σ_ii_*), and the mixing parameter (*k_ij_*). In this work, the pure fluid parameters are taken from previous works, where *n*-hexane is modeled as two tangent spheres [28], whereas water is modeled as a single sphere without electrostatic interactions [29]. In the latter case, the level of coarse graining averages out many important directional and long-range interactions present in water as a consequence of its very asymmetric molecular charge distribution. Such effects cannot be reproduced by a simplistic spherical isotropic potential, hence uniquely, the SAFT water molecular parameters are defined as a function of temperature and the type of properties (bulk or interfacial) that is targeted. In this work, we selected the set of molecular parameters based on interfacial tensions (see Table 1 for numerical values). Finally, for the alkane/water mixture, we used *k_ij_* = 0.3205 [33].

### 2.3. The Three-Phase Equilibrium from SAFT-VR Mie EoS

The SAFT-VR Mie EoS has been used as a thermodynamic model for describing the Helmholtz energy density of the homogeneous system (*a_0_*) and its derivate properties, such as chemical potential, *μ_i_*, and the stability function, ℑ. *a_0_* and *μ_i_* are needed to calculate the phase equilibrium condition as well as input to SGT, whereas ℑ is used to validate the phase equilibrium results. In this work, the three-phase equilibrium conditions in the canonical ensemble are described by the following expressions [34]:

(11a) $$-A_{V}\left(T,\rho^{L_{1}},x_{1}^{L_{1}}\right)=-A_{V}\left(T,\rho^{L_{2}},x_{1}^{L_{2}}\right)=-A_{V}\left(T,\rho^{V},x_{1}^{V}\right)$$

(11b) $$\mu_{1}\left(T,\rho^{L_{1}},x_{1}^{L_{1}}\right)=\mu_{1}\left(T,\rho^{L_{2}},x_{1}^{L_{2}}\right)=\mu_{1}\left(T,\rho^{V},x_{1}^{V}\right)$$

(11c) $$\mu_{2}\left(T,\rho^{L_{1}},x_{1}^{L_{1}}\right)=\mu_{2}\left(T,\rho^{L_{2}},x_{1}^{L_{2}}\right)=\mu_{2}\left(T,\rho^{V},x_{1}^{V}\right)$$

(11d) $$ℑ=A_{2x}A_{VV}-A_{x‘V}^{2}\geq 0$$

where the superscripts *L_1_*, *L_2_*, and *V* denote the three different bulk phases, *x_i_* and *μ_i_* are the mole fraction, and the chemical potential of component *i*, respectively. *A_nm_* is a shorthand notation for the derivative of *A* with respect to *n* and *m*. As an example, *A_V_* = (∂*A/*∂*V*), which gives the equilibrium pressure *P^0^* = - *A_V_*.

It is important to point out that Equation (11) are the necessary conditions of phase equilibrium for bulk phases. Specifically, Equation (11a) corresponds to the mechanical equilibrium condition (*P^L1^* = *P^L2^* = *P^V^* = *P^0^*) while Equations (11b) and (11c) express the chemical potential constraint (*μ_i_^L1^* = *μ_i_^L2^* = *μ_i_^V^*). Equation (11d) is a differential stability condition for phase equilibrium, comparable to the Gibbs energy stability constraint of a single phase [34]. Solving Equations (11a)–(11c) restricted to Equation (11d), it is possible to find the stable bulk densities and their mole fractions (*ρ^L1^*, *ρ^L2^*, *ρ^V^*, *x_1_^L1^*, *x_1_^L2^*, and *x_1_^V^*).

On the other hand, the cross-interaction parameter for SGT (*κ_12_*) is obtained by fitting Equation (1) with the experimental IFT values of the mixture [11]. The numerical value used in this work is *κ_12_* = 0.336, which is similar to the values reported by Cornelisse et al. [20] for the case of Peng-Robinson and APACT EoSs.

## 3. Molecular Dynamics Simulations

The molecular simulation methodology for the three-phase equilibrium has been described and applied in previous works for binary and ternary mixtures [18,19,23,24]. In this section, we retain the main technical aspects related to its initialization, equilibration, production as well as the calculation of the concentration profiles along three-phases interfacial region and their interfacial tensions. Some general considerations related to MD simulations to obtain interfacial tensions are recently described by Müller et al. [35] and Allen and Tildesley [36].

In general terms, the initial simulation cell to simulate multiphasic interfaces is similar to the traditional simulation cell used for biphasic systems, where one can initially equilibrate an independent box for each bulk phase and then assemble them in a single simulation box or alternatively use a single simulation box with the three fases distributed along *z* [35,36,37].

In this work, we use the latter approach where the of total number of molecules of the mixture, *N*, its distribution (*N* = *N_1_* + *N_2_*), and volume, *V,* at the simulation temperature, *T*, are estimated using the SAFT-VR Mie EoS, where the pure fluids and the fluid mixture are described by using the CG-Mie approach, i.e., the same Mie parameters taken from SAFT-VR Mie EoS (see Table 1). For a complete discussion of CG SAFT-VR Mie methodology and its top-down parameterization, the reader is directed to Müller and Jackson’s work [38].

Specifically, the MD simulations of the water + *n*-hexane binary mixture are performed using at least 19,000 Mie beads at the three-phase line conditions. The distribution of molecules (*N_1_*, *N_2_*) is fixed, and the systems are set up in such a way that the volume fractions of the resulting bulk phases are comparable. The simulations cell employs a *L_x_* × *L_y_* × *L_z_* parallelepiped with periodic boundary conditions in all three directions, with *L_x_ = L_y_ =* 55 Å (*L_x_ = L_y_ >* 10*σ*) and *L_z_* = 8 *L_x_* = 440 Å. This simulation box is built and filled with two liquid phases and a vapor phase assembled through the *z* axis (i.e.*,* the *L_1_*, *L_2_*, and V interfaces are located at the *xy* plane). In this assembly, each phase has an initial volume of *L_x_ = L_y_ =* 55, *Lz* = 145 Å, and is filled with a minimum of 19,000 Mie beads (in the whole simulation) at the three-phase line conditions. These values are chosen in order to have a large enough cell to accommodate two liquids, and the vapor regions with enough molecules to ensure a sensible statistics when calculating the densities of the bulk (non-interfacial) phase. To reduce the truncation and system size effects involved in the phase equilibrium and interfacial tension calculations, a cut-off radius of 27 Å (*r_cut_* ≈ 6*σ*) is used throughout this work.

All MD-CG simulations for phase equilibria and interfacial properties are performed using the LAMMPS code [39]. In this MD code, the rigid bond between the two tangential spheres of *n*-hexane is replaced by a harmonic potential of the from *U_bond_* = *K_b_* (*r* – *σ*)^2^, where *K_b_* = 7.583 kcal mol^−1^ Å^−2^ [33]. The velocity-Verlet integrator is used with a time step of 5 fs, and the temperature is controlled by a Nosé–Hoover thermostat with a relaxation constant of 0.2 ps.

First, this system is held at a high temperature, above its critical state (*T* > 1500 K), where a unique homogenous well-mixed phase is present. Then, the system is quenched at the desired temperature, and it is allowed to evolve under *NVT* conditions until equilibration is reached through diffusive mass transport [40]. After the initial temperature quenching, the systems are equilibrated for 500 ns. After this equilibration stage, a production run is set for at least another 500 ns. The corresponding statistics are accumulated extracted in block averages of 50 ns. Although a total time of 1000 ns might seem high a priori, it is important to note that three-phase systems tend to equilibrate slowler than the corresponding biphasic systems. In fact, the convergence of the total energy and density profiles of the triphasic system is monitored, determining that equilibration times between 100–300 ns are needed. For this reason, to ensure a true equilibration and a sensible production time 500+500 ns are chosen.

To characterize the bulk phase equilibria and interfacial properties, the concentration profiles, *ρ_i_*(*z*), are obtained by splitting the simulation box along the *z* direction in *L_x_* × *L_y_* × 1 Å^3^ bins and time averaging the number of molecules in each bin. Additionally, the center of mass of the system is fixed to its initial position to avoid profile smearing due to dynamical fluctuations. MD simulations allow evaluating the surface activity of species from the *ρ_i_*(*z*) profiles and the equilibrium pressure as well as the IFTs from Irving-Kirkwood method. Calculation of the two latter quantities requires obtaining the diagonal elements of the pressure tensor profiles along the direction of the box, which can be obtained in each bin using the virial expression [22,36]:

(12) $$P_{kk}=\kappa_{B}T\rho \left(z\right)+\frac{1}{S}\langle \overset{N-1}{\underset{i}{\sum }}\overset{N}{\underset{j>i}{\sum }}\frac{1}{\left|z_{i}-z_{j}\right|}{\left(f_{ij}\right)}_{k}{\left(r_{ij}\right)}_{k}\rangle$$

In Equation (12), *P_kk_* are the diagonal pressure tensor elements, where the subscript *k* represents the spatial coordinate, either *x*, *y*, or *z*. *k_B_* is Boltzmann’s constant, *T* is the absolute temperature, *S* is the interfacial area, *N* is the total number molecules, *f_ij_* is the force on molecule *i* due to molecule *j*, and *r_ij_* represents the distance between molecules *i* and *j*. *f_ij_* and *r_ij_* contributions have been equally distributed among the slabs corresponding to each molecule, and all the slabs between them. In Equation (12), the first term takes into account the kinetic contribution to the pressure, and it represents the perfect (ideal) gas term, while the second term corresponds to the configurational contribution, which is evaluated as ensemble averages, < >, and not at instant values.

From the pressure elements of Equation (12), one can extract the vapor pressure, corresponding to the *P_zz_* element, while the interfacial tension *γ*, between each pair of bulk phases *αβ* (i.e., *VL_1_*; *VL_2_* or *L_1_L_2_*) can be calculated by integrating the pressure elements of Equation (12) through the *z* dimension [21]:

(13) $$\gamma^{\alpha \beta }=\int_{z_{\alpha }}^{z_{\beta }}\left[P_{zz}\left(z\right)-\frac{P_{xx}\left(z\right)+P_{yy}\left(z\right)}{2}\right]dz$$

The three different tensions (*γ^VL1^*; *γ^VL2^*; *γ^L1L2^*) can be calculated from Equation (13) with the appropriate integration limits and are related to each other through the Antonow’s rule or the Neumann inequality.

As we established in the previous works [18,19,23,24], and following the standard procedure [35,37,41], to guarantee that the simulated systems are at a true equilibrium state, neither transient nor steady-state, we monitor the time evolution of the concentration profiles, the pressure tensor profiles, and the total energy of the system. Additionally, the fulfillment of the condition ∫ [*P_zz_*(*z*) – (*P_xx_*(z) + *P_yy_*(z))/2]*dz* = 0 within all bulk phases is verified.

**Figure 3:**
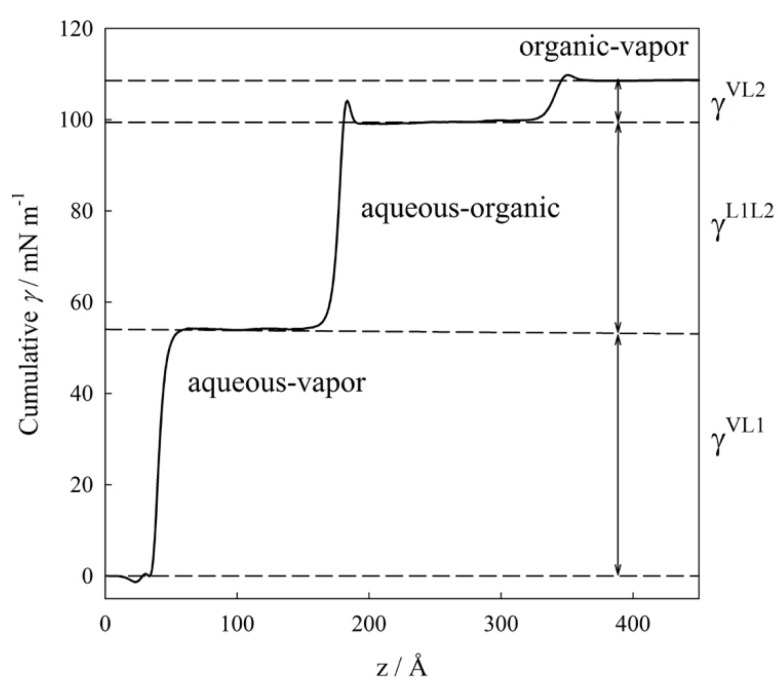
Cumulative interfacial tension, *γ,* as a function of the *z* coordinate for water (1) + *n*-hexane (2) mixture at the isothermal three-phase condition of 380 K. *γ^VL1^* ≈ 54.03 mN/m, *γ^L1L2^* ≈ (99.44–54.03) mN/m ≈ 46.41 mN/m, and *γ^VL2^* ≈ (108.56–99.44) mN/m ≈ 9.12 mN/m.

As an example, Figure 3 displays the variation of the interfacial tension as a function of the *z* coordinate for the isothermal condition of 380 K. From this figure it is possible to distinguish the three different regions where the IFT increases (i.e., the interfaces) and three plateaus (i.e., the bulk phases). From this plot, the IFT values are calculated as the change of IFT integral between two different plateaus. At this isothermal condition, *γ^VL1^* ≈ 54.03 mN/m, *γ^L1L2^* ≈ (99.44–54.03) mN/m ≈ 46.41 mN/m, and *γ^VL2^* ≈ (108.56–99.44) mN/m ≈ 9.12 mN/m. Further details related to the technical implementation of the previous expressions and their evaluation at three-phase conditions have been discussed in our previous works. [18,19,23,24]

## 4. Results and Discussions

The main aim of this work is to describe the interfacial properties (e.g., concentration profiles, surface activity, wetting transition, and interfacial tensions) along three-phase line for Type IIIa binary mixture, exemplified by the water + *n*-hexane mixture. In this section, we present the results and comparison of interfacial properties from the two methodologies used here (CG-MD and SGT coupled with the SAFT-VR Mie EoS), and also *T_w_* and *T_UCEP_* results are compared to the available experimental measurements [11,26,27].

### 4.1. Interfacial Tension Along a Three-Phase Equilibrium

**Figure 4:**
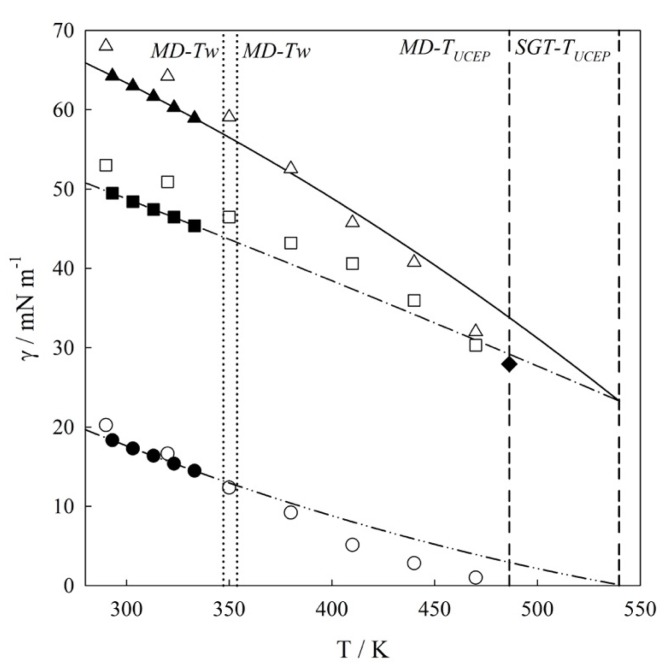
Interfacial tension, *γ*—temperature, *T* diagram for water (1) + *n*-hexane (2) mixture. Experimental data [11]: (▲) *γ^VL1^*; (■) *γ^L1L2^*; (●) *γ^VL2^*; MD results: (△) *γ^VL1^*; (☐) *γ^L1L2^*; (○) *γ^VL2^*; SGT + SAFT-VR Mie EoS calculations: (—) *γ^VL1^*; (– • –) *γ^L1L2^*; (– •• –) *γ^VL2^.* (•••) Estimated wetting temperature (MD-T_w_ = 347.20 K; SGT-T_w_ = 353.81 K); (- - -) Estimated Upper Critical End Point (UCEP) temperature (MD-T_UCEP_ = 486.30 K; SGT-T_UCEP_ = 539.78 K). (◆) *γ^UCEP^*.

The IFTs along the three-phase equilibrium are described by three aforementioned values *γ^VL1^*, *γ^VL2^* and *γ^L1L2^*. Figure 4 illustrates the thermal evolution of the interfacial tensions (*T* – *γ*) for the mixture at three-phase line. This figure includes the tensiometry data reported by Mori et al., [11], MD results (the numerical data are summarized in Table 2), and SGT + SAFT-VR Mie EoS calculations.

As seen in Figure 4, molecular simulations and theoretical calculations reproduce similar IFT thermal behavior as compared to experimental data within the experimental temperature range (293.15 K to 333.15 K). IFT experimental determinations for temperatures much higher than 333.15 K are not possible as they come close to and/or exceed the normal boiling point of *n*-hexane (*T_NB-C6H14_* = 341.88 K [25]). From Figure 4, an excellent agreement between SGT + SAFT-VR Mie EoS calculations and experimental data is observed. Noticeably, these theoretical calculations are not predictions as this agreement is a consequence of fitting the cross-influence parameter (*κ_ij_*). For the case of MD results, one observes an overprediction of IFT values but with low absolute average deviation; AAD*γ^VL1^* = 5%, AAD*γ^VL2^* = 7%; AAD*γ^L1L2^* = 7%. However, it is important to note that the tensiometry data reported by Mori *et al.,* [11] employed the density of the pure fluids rather than that of the mixture, and this choice influences the result inducing an *AADγ ≈* 2%, as was discussed by us in a previous work. (See Ref. [10] and references therein).

To explore the IFT at higher temperatures, both SGT and MD have been used. In this case, SGT results are extrapolations of a correlative model, whereas MD are fully predictive results. From Figure 4, it is also possible to observe that *γ^VL2^*→0, and *γ^VL1^* → *γ^L1L2^* when the mixture approaches its UCEP. As was described in Sec.2.1, this is the expected behavior of Type IIIa binary mixtures due to *ρ^L2^* ≈ *ρ^V^* as the UCEP is approached, then *γ^VL2^* ≃ 0, and *γ^VL1^* ≈ *γ^L1L2^*. Following the IFT extrapolations, the temperature of the UCEP (*T_UCEP_*) is estimated as 539.8 K from SGT, and 486.3 K from MD. Both results compare well with the value of 517 K calculated from theoretical predictions of interfacial tensions [20] and the experimental value 495.82 K [26]. The MD results suggest that *γ^VL1^* = *γ^L1L2^* = 27.93 mN/m at *T_UCEP_*. One possible reason for the overprediction of seen by the SGT is attributed to the fact that in general, while the SAFT-VR Mie model excels at predicting pure component critical points, it overestimates the critical condition of the mixtures [42]. The theoretical extrapolations are also in good agreement to ones reported by Cornelisse et al., [20] who calculated the IFTs from SGT combined to the Peng-Robinson and the APACT EoS.

The IFT values can be used to estimate the temperature of the wetting transition (*T_w_*), as the transition from equality to inequality in the relationship *γ^VL1^* ≤ *γ^L1L2^ + γ ^VL2^*. This mixture exhibits a wetting transition (*T_w_*) at 353.8 K (as estimated from SGT) and 347.2 K (as estimated from MD), which are both in close agreement with the value of 345.4 K reported by Bertrand et al. [27]. As it appears that *γ^VL1^* ≤ *γ^L1L2^ + γ ^VL2^* and using the criteria stated by Costas et al. [8], it is possible to conclude that the wetting transition is of first order.

### 4.2. Bulk Densities and Interfacial Concentration Profiles Along a Three-Phase Equilibrium

The concentration of species (*ρ_i_*) along the interfacial region (*z*) for three-phase system is characterized by three bulk zones and their interfaces: *VL_1_*, *VL_2_*, and *L_1_L_2_*, as was schematically illustrated in Figure 2d. The *z* – *ρ_i_* diagrams are used to describe the interfacial concentration, the surface activity of species, and the wetting behavior. In this section, the *z* – *ρ*_i_ diagrams are described at three selected isothermal conditions: (i) *T* < *T_w_*, (ii) *T* > *T_w_*, (iii) *T* ≈ *T_UCEP_*.

Table 3 summarizes the equilibrium bulk densities and mole fractions obtained from MD. From this table, it is evident that the aqueous bulk phase is, statistically, pure water. The organic bulk phase is rich in *n*-hexane with some water and the vapor phase is rich in water (70 to 80%). This anomalous high concentration of water in the vapor phase can be attributed to the selected CG model for water, which was originally fit to reproduce the interfacial tension rather than vapor pressure. The MD results of the bulk densities follow the expected *T* – *ρ* projection of a Type IIIa illustrated in Figure 1, where the extrapolated conditions, using scaling laws [34], for the UCEP are *T_UCEP_* = 479.10 K, and *ρ_UCEP_* = 0.2667 g cm^−3^, which show good agreement with the experimental values (495.82 K and 0.2599 g cm^−3^ [26]). Additionally, the extrapolated *T_UCEP_* (from bulk phase calculations) agrees with those obtained from IFTs. Table 3 includes the corresponding extrapolated conditions for the UCEP.

**Figure 5:**
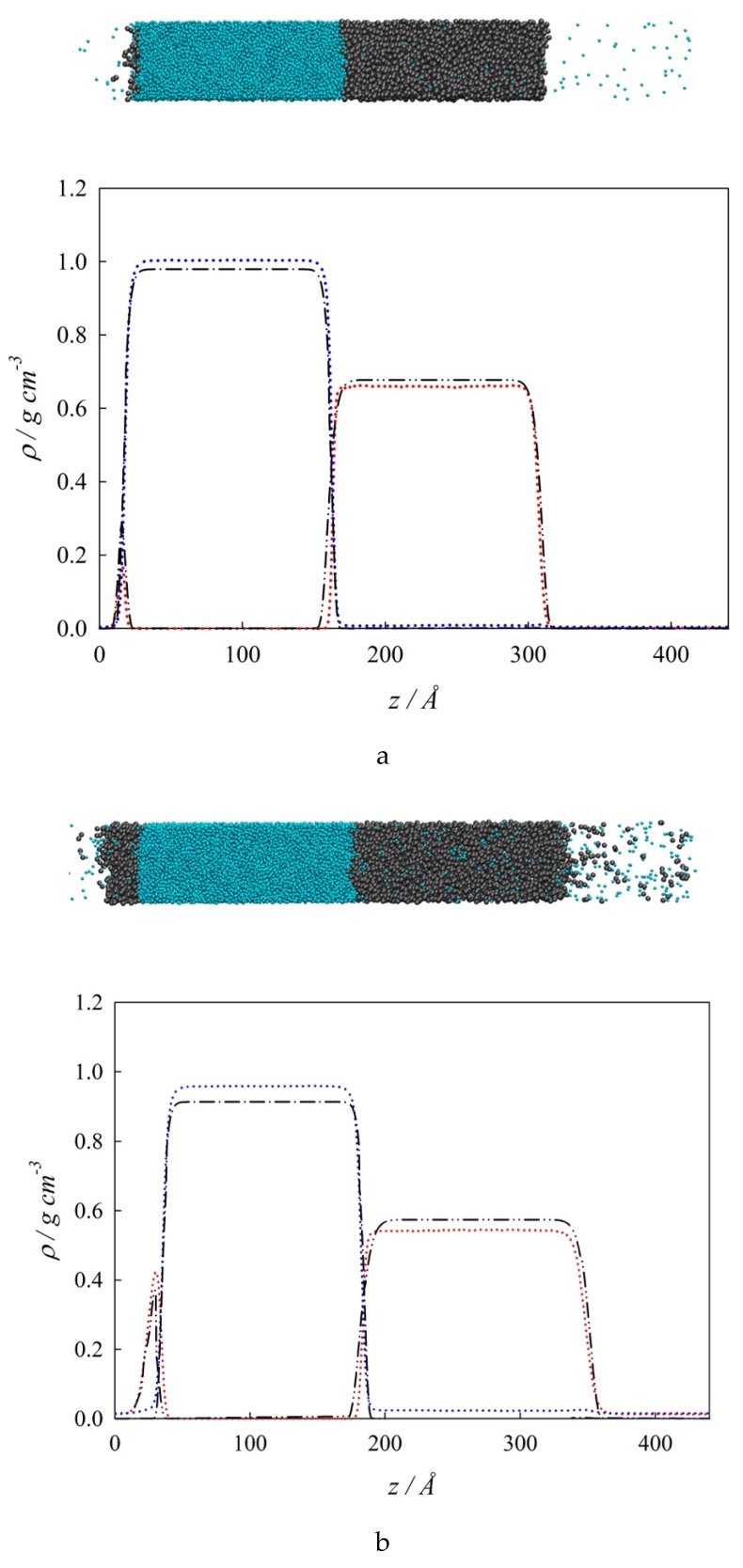

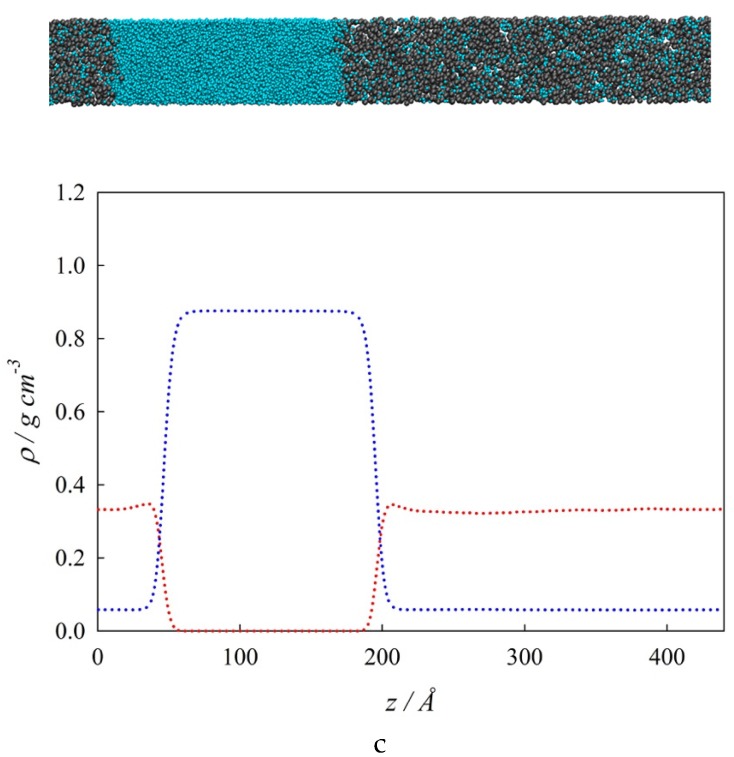
Interfacial concentration distribution along the interfacial region at three different isothermal conditions. (**a**). 290 K; (**b**). 380 K; (**c**). 485 K. Top: Snapshot: (●) water, (●●) *n*-hexane. Bottom: Interfacial concentration profiles, *ρ_i_,* along the interfacial region, *z.* SGT + SAFT-VR Mie EoS calculations: (– • –) water, (– •• –) *n*-hexane. MD results: (•••) water, (•••) *n*-hexane.

For the case of interfacial concentration profiles, Figure 5 show both a snapshot (top) and the corresponding concentration profiles (bottom) at three selected temperatures. Specifically, Figure 5a collects the MD and SGT results for the *z* – *ρ*_i_ profiles at the isothermal condition of partial wetting of 290 K (*T* < *T_w_*). The concentration profiles in this figure reveal that both MD simulations and SGT results are in very good agreement with each other. Focusing on the *VL_1_* interfacial behavior (i.e., close to *z* = 0 and left hand in the insert snapshot) the aqueous bulk liquid region (*L_1_*) and the bulk vapor region (*V*) are partially separated by an organic bulk liquid region (*L_2_*). This type of structure is a clear evidence that *L_2_* partially wets the *VL_1_* interface at this thermodynamic condition. In other words, the interfacial concentration profile that connects the bulk phases *VL_1_* is different than the sum of the *L_1_L_2_* and the *VL_2_* interfacial concentration profiles (i.e., *ρ^VL1^*(*z*) ≠ *ρ^L1L2^ (z*) + *ρ^VL2^* (*z*)). It can also be observed that the density of *n*-hexane shows a high peak that reflects a strong positive surface activity (accumulation or adsorption) at the *VL_1_* interface, whereas the density of water displays a weak positive surface activity at the *VL_2_*.

Figure 5b showcases the *z* – *ρ*_i_ profiles at the isothermal condition total wetting of 380 K (*T* > *T_w_*). Similar to Figure 5a, Figure 5b displays a very good agreement between MD simulations and SGT results. Positive surface activity of *n*-hexane and water is observed at the *VL_1_* and *VL_2_* interfaces, respectively. It is important to point out that the surface activity of *n*-hexane notoriously increases from 290 K to 380 K. From Figure 5b, it is possible to observe that as *L_2_* completely wets the *VL_1_* interface at these thermodynamic conditions, then *ρ^VL1^*(*z*) = *ρ^L1L2^* (*z*) + *ρ^VL2^* (*z*). Finally, Figure 5c shows the *z* – *ρ_i_* profiles and a snapshot of the system at an isothermal condition of 485 K, which is near the *T_UCEP_*. At this temperature, *ρ^L2^* ≈ *ρ^V^*, and *ρ^L1^* ≠ 0 which means that only *VL_1_* interfaces are observed. Additionally, the surface activity of *n*-hexane is still noticeable, whereas water displays no surface activity. This figure includes the MD results only because the SGT predicts three phases at this temperature.

## 5. Conclusions

The main aim of this work is the description of interfacial properties, interfacial profiles, wetting behavior and interfacial tensions along the three-phase equilibrium line for a Type IIIa binary mixture. This type of system is exemplified by the water + *n*-hexane mixture which is described by CG SAFT intermolecular potentials parametrized using a top-down approach. The calculations are based on MD simulations and a theoretical approach which applies the SGT coupled to the SAFT-VR Mie EoS. Both approaches show a good agreement with each other, and their interfacial tension results are comparable to the available tensiometric determinations.

Based on the results, a first order wetting transition at 353.8 K is estimated from SGT, whereas MD estimates a value of 347.2 K, which is close to the reported value of 345.4 K. This transition from partial to total wetting is also demonstrated in the interfacial concentration profiles and interfacial tensions. Additionally, the extrapolation of the interfacial tension results provides a route to estimate the UCEP temperature. SGT+SAFT-VR Mie extrapolates this condition at 539.8 K, and MD suggests an answer in the range of 479.1 K to 486.3 K, which also are close to the literature values of 495.82 K. The combination of MD and SGT provides the concentration profiles along the interfacial region, and the surface activity of hexane and water. From these profiles, it is observed that *n*-hexane surface activity increases and water surface decreases with temperature.

The good agreement between both approaches and the systematic way of obtaining the molecular parameters used to perform this study highlights the main advantages of the proposed methodology: (a) the use of the same molecular parameters for both MD simulations and SGT + SAFT-VR Mie calculations provide a *vis à vis* comparison, (b) MD simulations can be used to validate the SGT + SAFT-VR Mie calculations, and (c) the right combination of an appropriate EoS, SGT, and MD provides a robust methodology for the description of interfacial properties along the three-phase equilibrium line.

## Figures and Tables

**Table 1:** Coarse-Grained Mie parameters for pure components ^a,b,c^.

*Fluid*	m_*si*_	λ_*r,ii*_	*ε*_*ii*_*/k*_B_/K	*σ*_*ii*_/Å
*n*-hexane (*n*-C_6_H_14_)	2	19.57	376.35	4.508
water (H_2_O)	1	8.00	−4.806 × 10^−4^ T^2^ + 0.6107 T + 165.9	−6.455 × 10^−9^ T^3^ + 9.1 x 10^−6^ T^2^ − 4.291 x 10^−3^ T + 3.543

^a^ The pure fluid Mie parameters are taken from Mejía et al. [28] for *n*-hexane, and Lobanova et al. [29] for water, T in K. ^b^ The attractive exponent was fixed at 6 (*λ_a,ii_* = 6) for both pure fluids. ^c^ The influence parameters (*c_ii_*) of pure fluids for SGT are calculated from Equation (2).

**Table 2:** Interfacial tensions result from Molecular Dynamics for water (1) + *n*-hexane (2) along three-phase equilibrium ^a,b^.

*T/K*	γ^*VL1*^*/mN m*^*−1*^	γ^*L1L2*^*/mN m*^*−1*^	γ^*VL2*^*/mN m*^*−1*^
290	68.00_1_	53.00_1_	20.24_1_
320	64.20_1_	50.90_1_	16.64_3_
350	59.06_2_	46.47_3_	12.36_2_
380	54.68_3_	43.20_2_	9.20_2_
410	45.74_3_	40.61_2_	5.13_4_
440	40.75_1_	35.96_5_	2.83_3_
470	32.05_2_	30.30_3_	1.00_2_
486.3^ c^	27.93	27.93	0.00

^a^ The subscripted number is the uncertainty in the last digits. (i.e., 20.24_1_ means 20.24 ± 0.01). ^b^ *L_1_*: aqueous (water) rich phase; *L_2_*: oil (*n*-hexane) rich phase; *V*: vapor phase. ^c^ Extrapolated upper critical conditions.

**Table 3:** Mass bulk densities and mole fractions results from Molecular Dynamics for water (1) + *n*-hexane (2) mixture along three-phase equilibrium ^a^.

Organic (*n*-hexane rich) phase				
*T/K*	*x_1_*	*ρ_1_/g cm^−3^*	*ρ_2_/g cm^−3^*	*ρ/g cm^−3^*
290	0.047_1_	0.0071_1_	0.661_2_	0.668_3_
320	0.081_1_	0.0121_1_	0.626_3_	0.638_3_
350	0.116_2_	0.0163_3_	0.587_1_	0.603_2_
380	0.169_1_	0.0233_2_	0.543_4_	0.566_2_
410	0.232_3_	0.0312_2_	0.489_1_	0.520_3_
440	0.338_1_	0.0442_1_	0.412_2_	0.456_3_
470	0.455_4_	0.0581_2_	0.331_1_	0.389_1_
479.10 ^b^	0.501	0.134	0.134	0.268
Aqueous (water rich) phase				
*T/K*	*x_1_*	*ρ_1_/g cm^−3^*	*ρ_2_/g cm^−3^*	*ρ/g cm^−3^*
290	1.000	1.003_1_	0.000	1.003_1_
320	1.000	0.994_2_	0.000	0.994_2_
350	1.000	0.979_2_	0.000	0.979_2_
380	1.000	0.958_1_	0.000	0.958_1_
410	1.000	0.934_3_	0.000	0.934_3_
440	1.000	0.905_1_	0.000	0.905_1_
470	1.000	0.875_2_	0.000	0.875_2_
479.1^b^	1.000	0.864	0.000	0.864
vapor phase				
*T/K*	*x_1_*	*ρ_1_/g cm^−3^*	*ρ_2_/g cm^−3^*	*ρ/g cm^−3^*
290	0.964_1_	0.004_1_	0.001_1_	0.005_1_
320	0.925_1_	0.006_1_	0.003_1_	0.009_1_
350	0.901_2_	0.010_2_	0.006_3_	0.016_2_
380	0.849_1_	0.015_2_	0.013_2_	0.028_2_
410	0.807_3_	0.024_1_	0.027_2_	0.051_1_
440	0.755_1_	0.034_3_	0.053_2_	0.119_2_
470	0.571_3_	0.127_4_	0.095_6_	0.223_1_
479.1 ^b^	0.501	0.134	0.134	0.268

^a^ The subscripted number is the uncertainty in the last digits. (i.e., 0.661_2_ means 0.661 ± 0.002). ^b^ Extrapolated upper critical conditions.

## References

[1] Van Konynenburg P., Scott R. (1980). Critical lines and phase equilibria in binary van der Waals mixtures. Philos. Trans. R. Soc..

[2] Bidart C., Segura H., Wisniak J. (2007). Phase equilibrium behavior in water (1) + *n*-alkane (2) mixtures. Ind. Eng. Chem. Res..

[3] Virnau P., Müller M., MacDowell L.G., Binder K. (2004). Phase behavior of n-alkanes in supercritical solution: A Monte Carlo study. J. Chem. Phys..

[4] Müller M., MacDowell L.G., Virnau P., Binder K. (2002). Interface properties and bubble nucleation in compressible mixtures containing polymers. J. Chem. Phys..

[5] Rowlinson J.S., Widom B. (1998). Molecular Theory of Capillarity.

[6] Cahn J.W. (1977). Critical point wetting. J. Chem. Phys..

[7] Dietrich S., Latz A. (1989). Classification of interfacial wetting behavior in binary liquid mixtures. Phys. Rev. B.

[8] Costas M.E., Varea C., Robledo A. (1983). Global phase diagram for the wetting transition at interfaces in fluid mixtures. Phys. Rew. Lett..

[9] Evans M.J.B., Weir R.D.D., de Loos T.W.W. (2005). Measurement of Surface and Interfacial Tension. Measurement of the Thermodynamic Properties of Multiple Phases.

[10] Mejía A., Cartes M., Segura H., Müller E.A. (2014). Use of equations of state and coarse grained simulations to complement experiments: Describing the interfacial properties of carbon dioxide + decane and carbon dioxide + eicosane mixtures. J. Chem. Eng. Data.

[11] Mori Y.H., Tsul N., Klyomlya M. (1984). Surface and interfacial tensions and their combined properties in seven binary, immiscible liquid-liquid-vapor systems. J. Chem. Eng. Data.

[12] Van der Waals J.D. (1893). Thermodynamische Theorie der Kapillarität unter voraussetzung Stetiger dichteänderung. Zeit. Phys. Chem..

[13] Carey B.S. (1979). The Gradient Theory of Fluid Interfaces. Ph.D. Thesis.

[14] Telo da Gama M.M., Evans R. (1983). The structure and surface tension of the liquid-vapour interface near the upper critical end point of a binary mixture of Lennard-Jones fluids: I. The two phase region. Mol. Phys..

[15] Tarazona P., Telo da Gama M.M., Evans R. (1983). Wetting transitions at fluid-fluid interfaces: I. The order of the transition. Mol. Phys..

[16] Mejía A., Segura H. (2004). Interfacial behavior in systems Type IV. Int. J. Thermophys..

[17] Mejía A., Segura H. (2005). On the interfacial behavior about the shield region. Int. J. Thermophys..

[18] Mejía A., Pàmies J.C., Duque D., Segura H., Vega L.F. (2005). Phase and interface behavior in type I and type V Lennard-Jones mixtures: Theory and Simulations. J. Chem. Phys..

[19] Mejía A., Vega L.F. (2006). Perfect wetting along a three-phase line: Theory and molecular dynamics simulations. J. Chem. Phys..

[20] Cornelisse P.M.W., Peters C.J., de Swaan Arons J. (1998). Interfacial phase transitions at solid-fluid and liquid-vapor interfaces. Int. J. Thermophys..

[21] Hulshof H. (1901). Ueber die Oberflächenspannung. Ann. Phys..

[22] Irving J.H., Kirkwood J.G. (1950). The statistical mechanical theory of transport processes. IV. The equations of hydrodynamics. J. Chem. Phys..

[23] Garrido J., Quinteros-Lama H., Piñeiro M.M., Mejía A., Segura H. (2014). On the phase and interface behavior along the three-phase line of ternary Lennard-Jones mixtures. A collaborative approach based on square gradient theory and molecular dynamics simulations. J. Chem. Phys..

[24] Müller E.A., Mejía A. (2014). Resolving discrepancies in the measurements of the interfacial tension for the CO_2_ + H_2_O mixture by computer simulation. J. Phys. Chem. Lett..

[25] Daubert T.E., Danner R.P. (1989). Physical and Thermodynamic Properties of Pure Chemicals. Data Compilation.

[26] Kamilov I.K., Stepanov G.V., Abdulagatov I.M., Rasulov A.R., Milikhina E.I. (2001). Liquid-liquid-vapor, liquid-liquid, and liquid-vapor phase transitions in aqueous n-hexane mixtures from isochoric heat capacity measurements. J. Chem. Eng. Data.

[27] Bertrand E., Dobbs H., Broseta D., Indekeu J., Bonn D., Meunier J. (2000). First-order and critical wetting of alkanes on water. Phys. Rev. Lett..

[28] Mejía A., Herdes-Moreno C., Müller E.A. (2014). Force fields for coarse-grained molecular simulations from a corresponding states correlation. Ind. Eng. Chem. Res..

[29] Lobanova O., Avendaño C., Lafitte T., Müller E.A., Jackson G. (2015). SAFT-γ force field for the simulation of molecular fluids: 4. A single-site coarse-grained model of water applicable over a wide temperature range. Mol. Phys..

[30] Lafitte T., Apostolakou A., Avendaño C., Galindo A., Adjiman C.S., Müller E.A., Jackson G. (2013). Accurate statistical associating fluid theory for chain molecules formed from Mie segments. J. Chem Phys..

[31] Garrido J.M., Piñeiro M.M., Blas F.J., Müller E.A., Mejía A. (2016). Interfacial tensions of industrial fluids from a molecular-based square gradient theory. AIChE J..

[32] Mie G. (1903). Zur kinetischen Theorie der einatomigen Körper. Ann. Phys..

[33] Lobanova O. (2014). Development of Coarse-Grained Force Fields from a Molecular Based Equation of State for Thermodynamic and Structural Properties of Complex Fluids. Ph.D. Thesis.

[34] Rowlinson J.S., Swinton F. (1982). Liquids and Liquids Mixtures.

[35] Müller E.A., Ervik Å., Mejía A. Best Practices for Computing Interfacial Properties from Molecular Dynamics Simulations. https://www.livecomsjournal.org.

[36] Allen M.P., Tildesley D.J. (2017). Computer Simulation of Liquids.

[37] Holcomb C.D., Clancy P., Zollweg J.A. (1993). A critical study of the simulation of the liquid-vapour interface of a Lennard-Jones fluid. Mol. Phys..

[38] Müller E.A., Jackson G. (2014). Force-field parameters from the SAFT-γ equation of state for use in coarse-grained molecular simulations. Annu. Rev. Chem. Biomol. Eng..

[39] Plimpton S. (1995). Fast parallel algorithms for short-range molecular dynamics. J. Comput. Phys..

[40] Martinez-Veracoechea F., Müller E.A. (2005). Temperature-quench molecular dynamics simulations for fluid phase equilibria. Mol. Simulat..

[41] Duque D., Vega L.F. (2004). Some issues on the calculation of interfacial properties by molecular simulation. J. Chem. Phys..

[42] Aasen A., Hammer M., Müller E.A., Wilhelmsen Ø. (2020). Equation of state and force fields for Feynman–Hibbs-corrected Mie fluids. II. Application to mixtures of helium, neon, hydrogen, and deuterium. J. Chem. Phys.

